# Early Recalls and Clinical Validation Gaps in Artificial Intelligence–Enabled Medical Devices

**DOI:** 10.1001/jamahealthforum.2025.3172

**Published:** 2025-08-22

**Authors:** Branden Lee, Patrick Kramer, Sara Sandri, Ritika Chanda, Crystal Favorito, Olivia Nasef, Joseph S. Ross, Joshua Sharfstein, Tinglong Dai

**Affiliations:** 1 Department of Orthopaedic Surgery, Johns Hopkins Medicine, Baltimore, Maryland; 2 School of Medicine, Johns Hopkins University, Baltimore, Maryland; 3 Bloomberg School of Public Health, Johns Hopkins University, Baltimore, Maryland; 4 School of Medicine, Georgetown University, Washington, District of Columbia; 5 Department of Internal Medicine, Yale School of Medicine, New Haven, Connecticut; 6 Department of Health Policy and Management, Yale School of Public Health, New Haven, Connecticut; 7 Carey Business School, Johns Hopkins University, Baltimore, Maryland; 8 Hopkins Business of Health Initiative, Johns Hopkins University, Washington, DC

## Abstract

This cross-sectional study examines whether lack of clinical validation and publicly traded manufacturer status were associated with recalls of artificial intelligence–enabled medical devices that had been cleared by the US Food and Drug Administration.

## Introduction

Artificial intelligence–enabled medical devices (AIMDs) are increasingly present in US clinical practice,^1^ with nearly all cleared through the US Food and Drug Administration (FDA) 510(k) pathway.^2,3,4^ Because 510(k) clearance does not require prospective human testing, many AIMDs enter the market with limited or no clinical evaluation; meanwhile, recalls may undermine clinician and patient confidence in their performance. We examined whether lack of reported clinical validation and publicly traded manufacturer status were associated with AIMD recalls.

## Methods

In this cross-sectional study, all FDA-cleared AIMDs^1^ were matched to recall entries in the Center for Devices and Radiological Health database from November 15 to November 30, 2024. Each recall number–device pair was counted as 1 event; devices could contribute multiple events. Validation status was classified as none, retrospective dataset, or prospective trial based on FDA summaries. AIMD commercialization models were determined using S&P Capital IQ NetAdvantage and PrivCo databases using previously described schema.^5^ Manufacturer-attributed recall causes were categorized as diagnostic or measurement errors, functionality delay or loss, physical hazards, biochemical hazards, and postmarket changes. We followed the STROBE reporting guideline.

Kaplan-Meier analyses estimated recall-free survival; group differences were assessed using log-rank tests. Multivariable logistic regression (GraphPad Prism 10; 2-sided α = .05) modeled the odds of any recall, adjusting for validation status, manufacturer type, clearance year (2020-2024 vs earlier), and clinical specialty. Because the study used publicly available data, it did not require institutional review board oversight or informed consent per the Common Rule (US 45 CFR 46).

## Results

**Figure 1.**
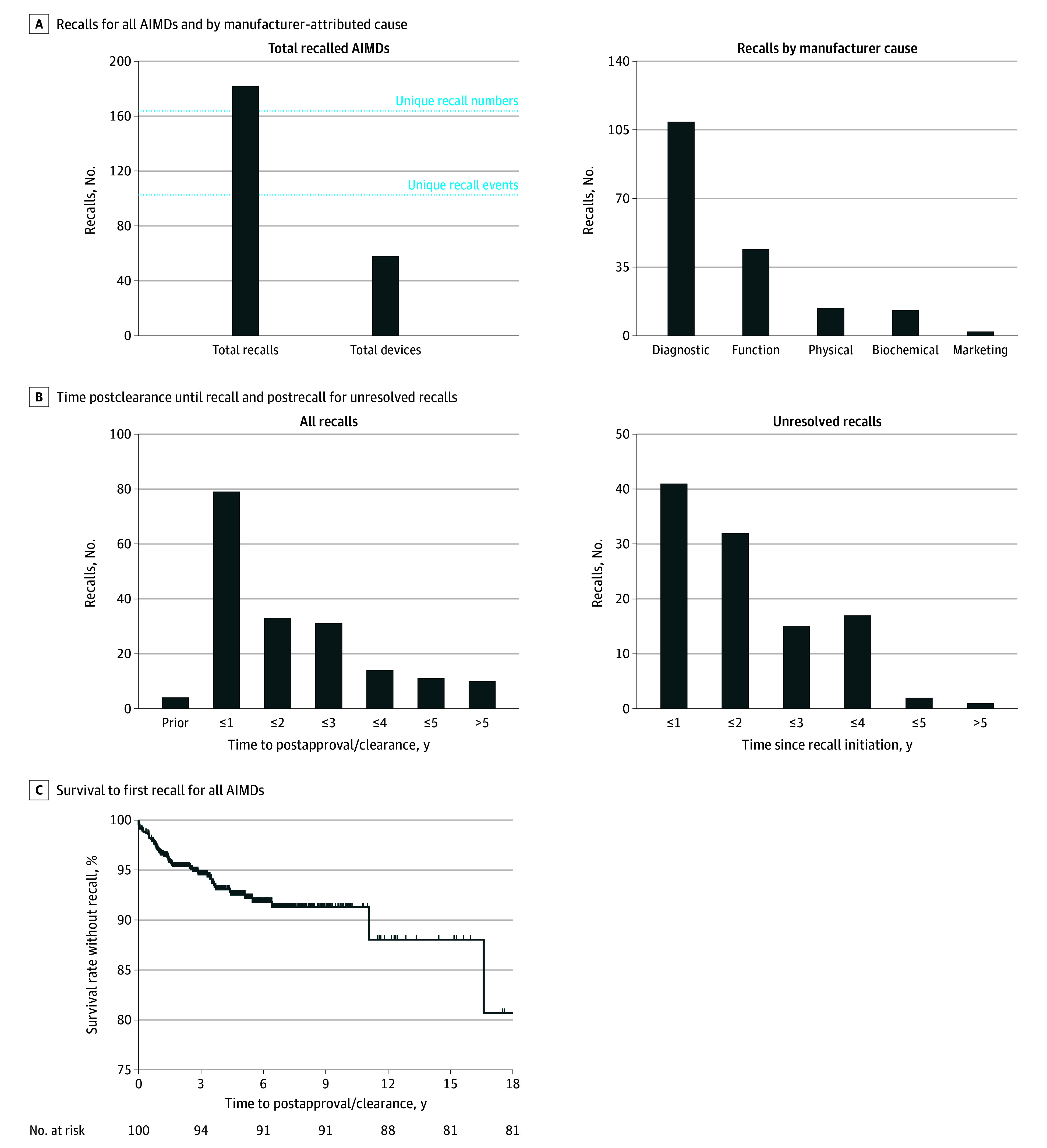
Overview of Artificial Intelligence-Enabled Medical Device (AIMD) Recalls A, Total number of recalls (n = 182) and AIMDs recalled (n = 60) for all recalled AIMDs (left) and by manufacturer-attributed causes of recall (right). In the left panel, the total recalls represent each US Food and Drug Administration (FDA) recall number–device pairing, the unique recall numbers represent the distinct FDA-issued recall IDs, the unique recall events represent recall numbers arising from 1 issue, and the total devices represent AIMDs with 1 or more recalls. B, Time elapsed between device clearance and recall initiation (left) and since recall initiation for nonterminated recalls (right). Each bin is a 1-year interval up to 5 years. C, Kaplan-Meier survival curve showing recall-free survival over time since FDA clearance.

Among 950 AIMDs, 60 (6.3%) were associated with 182 recall events (mean [SD] recalls per device, 3.0 [4.3]) (Figure 1A). Diagnostic or measurement errors accounted for 109 recalls encompassing 935 063 units, followed by functionality delay (44 recalls, 755 647 units), physical hazards (14 recalls, 8192 units), and biochemical hazards (13 recalls, 76 257 units). Of these recalls, 79 (43.4%) occurred within the first 12 months of device clearance (Figure 1B), approximately double the rate reported for all 510(k) devices.^4^ Notably, 108 recalls (59.3%) remained unresolved at the study end date, with 20 recalls remaining unresolved for more than 3 years. Overall, recall-free survival was 96.6% at 1 year, 93.5% at 3 years, and 91.8% at 5 years (Figure 1C).

**Figure 2.**
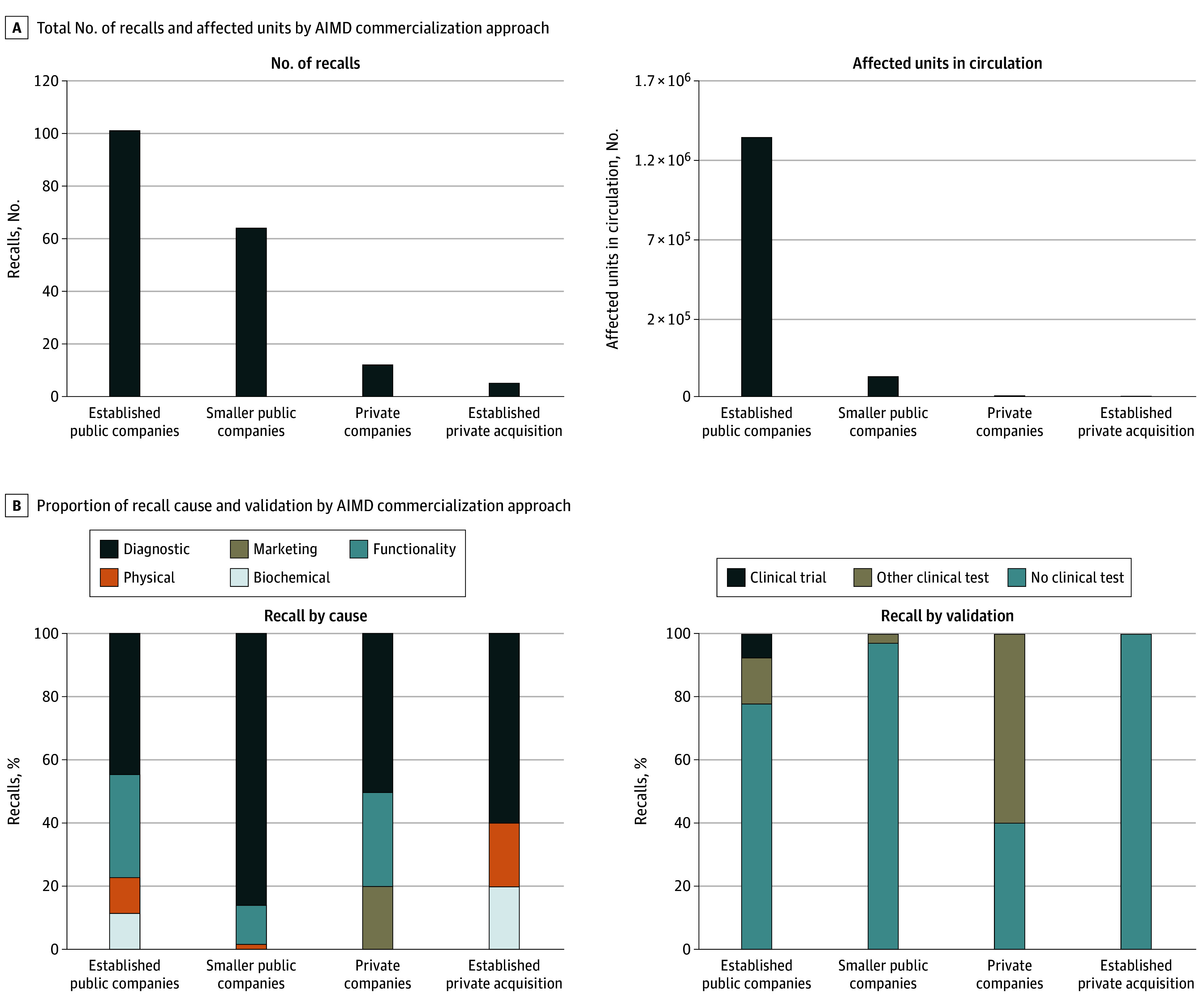
Recall Counts, Cause, and Affected Units by Commercialization Model A, Total number of recalled devices (left) and affected units (right) across commercialization models. For the segment break at 10 000 units, the scale below the segmentation is 5000 per tick mark, and the scale above the segmentation is 200 000 units per tick mark. B, Proportions of recall causes for recalled devices (left) and by differing degrees of clinical testing (right) across commercialization models.

Devices without reported validation had more recalls per device (mean [SD], 3.4 [5.1]) than those with retrospective (1.9 [1.5]) or prospective (2.0 [1.4]) validation, and were associated with larger recalls (mean [SD] units recalled, 12 193 [82 405] vs 6523 [18 690]; *P* < .001). Public companies accounted for 53.2% of AIMDs but 91.8% of recalls and 98.7% of recalled units (Figure 2A). Among recalled devices from privately traded companies, 40% lacked clinical validation compared with 77.7% of those from established public companies and 96.9% from smaller public companies (Figure 2B). Multivariable analysis revealed that lack of clinical validation (odds ratio [OR], 2.8; 95% CI, 1.6-4.7) and public company status (OR, 5.9; 95% CI, 2.4-14.6) were independently associated with recall.

## Discussion

In this cross-sectional study, recalls of FDA-cleared AIMDs were uncommon but were concentrated early after clearance and predominantly involved products lacking clinical validation and manufactured by publicly traded companies, suggesting that the 510(k) process may overlook early performance failures of AI technologies. Requiring prospective evaluation or issuing time-limited clearances that lapse without confirmatory data may reduce these risks.^2,3,4^ Given that over 90% of recalled units were produced by public companies, heightened premarket clinical testing requirements and postmarket surveillance measures may improve identification and reduction of device errors, similar to risk-based strategies in pharmacovigilance.^6^ The association between public company status and higher recalls may reflect investor-driven pressure for faster launches, warranting further study.

Study limitations include reliance on publicly available validation reports, exclusion of software updates not labeled as recalls, and limited follow-up for devices cleared after 2022. Nevertheless, linking premarket evidence gaps and manufacturer type to postmarket actions offers practical guidance for regulators, clinicians, and health systems adopting AI-based tools.

## Notes

Supplement.
Data Sharing Statement
