# Supplementary material for: Early Recalls and Clinical Validation Gaps in Artificial Intelligence–Enabled Medical Devices
Source: JAMA Health Forum. 2025 Aug 22;6(8):e253172. doi: 10.1001/jamahealthforum.2025.3172 (PMC12374217; doi:10.1001/jamahealthforum.2025.3172)
Supplement: Supplement. — Data Sharing Statement [file jamahealthforum-e253172-s001.pdf]

Data Sharing Statement

Lee. Early Recalls and Clinical Validation Gaps in Artificial Intelligence–Enabled Medical Devices. *JAMA Health Forum*. Published August 22, 2025.
doi:10.1001/jamahealthforum.2025.3172

Data
Data available: Yes
Data types: Data (not involving human participants)
How to access data: Data will be available upon reasonable request to dai@jhu.edu
When available: With publication

Supporting Documents
Document types: None

Additional Information
Who can access the data: Researchers whose proposed use of the data has been approved.
Types of analyses: For research purposes related to AI medical device safety, regulatory oversight, or post-market surveillance.
Mechanisms of data availability: After approval of a proposal and with a signed data access agreement.
